# ^1^H, ^15^N, ^13^C resonance assignment of human GAP-43

**DOI:** 10.1007/s12104-015-9660-9

**Published:** 2016-01-09

**Authors:** Andrea Gabriele Flamm, Szymon Żerko, Anna Zawadzka-Kazimierczuk, Wiktor Koźmiński, Robert Konrat, Nicolas Coudevylle

**Affiliations:** Department of Computational and Structural Biology, Max F. Perutz Laboratories, University of Vienna, Campus Vienna Biocenter 5, 1030 Vienna, Austria; Faculty of Chemistry, Biological and Chemical Research Centre, University of Warsaw, Żwirki i Wigury 101, 02-089 Warsaw, Poland

**Keywords:** GAP-43, Neuromodulin, Intrinsically disordered protein, Growth cone

## Abstract

GAP-43 is a 25 kDa neuronal intrinsically disordered protein, highly abundant in the neuronal growth cone during development and regeneration. The exact molecular function(s) of GAP-43 remains unclear but it appears to be involved in growth cone guidance and actin cytoskeleton organization. Therefore, GAP-43 seems to play an important role in neurotransmitter vesicle fusion and recycling, long-term potentiation, spatial memory formation and learning. Here we report the nearly complete assignment of recombinant human GAP-43.

**Electronic supplementary material:**

The online version of this article (doi:10.1007/s12104-015-9660-9) contains supplementary material, which is available to authorized users.

## Biological context

GAP-43 is a 25 kDa neuronal intrinsically disordered protein (IDP) highly abundant in the neuronal growth cone during development and regeneration. The exact molecular function(s) of GAP-43 remains unclear but it appears to be involved in growth cone guidance and actin cytoskeleton organization (Frey et al. 2000). Therefore, GAP-43 seems to play an important role in neurotransmitter vesicle fusion and recycling, long-term potentiation, spatial memory formation and learning (Denny 2006). GAP-43 experiences S-palmitoylation on positions Cys3 and Cys4 (Liu et al. 1993). Once acylated, it is bound to the inner leaflet of the plasma membrane in the growth cone of axons where it sequesters phosphatidylinositol-4,5-bisphosphate (PIP2) into lipid rafts via electrostatic interactions involving polybasic stretches (Laux et al. 2000). GAP-43 appears to be regulated by calcium- and PIP2-signaling cascades as it can bind calmodulin (CaM) and is the substrate of protein kinase C (PKC) (Apel et al. 1990; Maekawa et al. 1994). GAP-43 interacts with both apo and holo-CaM via its IQ domain (from His32 to Leu51), which adopts a helical conformation upon binding (Kumar et al. 2013). Phosphorylation by PKC occurs within the IQ domains at Ser41. Phosphorylation prevents the association with CaM and influences membrane binding (Maekawa et al. 1994; Tejero-Diez et al. 2000). Additionally, the phosphorylated form of GAP-43 seems to stabilize actin filaments via a direct interaction (He et al. 1997). Interestingly, GAP-43 is functionally related to the neuronal IDP BASP1, which is also under investigation in our group.

In order to gain insight into the intriguing membrane and ligand binding properties of GAP-43 and how post-translational modifications affect its structural dynamics and therefore its binding properties, we first achieved the near complete chemical shift assignment of human GAP-43.

## Methods and results

### Protein expression and purification

The coding region for human GAP-43 was obtained by assembly PCR (Stemmer et al. 1995), this procedure allows to directly optimize the coding sequence for the chosen expression system and to avoid undesired restriction sites inside the coding sequence. 32 oligonucleotides, covering the entire coding sequence of GAP-43, were designed using the online software DNAworks (Hoover and Lubkowski 2002). The PCR gene assembly was realized according to a published protocol (Stemmer et al. 1995). The final gene amplification was performed from 1 μl of the gene assembly mixture by introducing 5′ NdeI and 3′ NotI restriction sites. Subsequently, the fragment was inserted in-frame into the NdeI and NotI sites of the bacterial expression vector pET-41b, yielding pET-41b-hGAP-43, encoding human GAP-43 fused to a C-terminal His6-tag. The two cysteines on position 3 and 4 were replaced by glycines by site-directed mutagenesis. ^15^N/^13^C labeled GAP-43 was expressed in the *Escherichia coli* strain T7 express (New England BioLab) in minimal medium containing ^15^N-labeled ammonium chloride and ^13^C-glucose as sole nitrogen and carbon sources, respectively. GAP-43 expression was induced at an *A*_600nm_ of 0.6 by addition of 0.8 mM IPTG. The cells were collected after 16 h of expression at 30 °C by centrifugation at 6000 rpm for 10 min and resuspended in 30 ml of ice-cold lysis buffer (20 mM NaP_i_ pH 7.4, 50 mM NaCl) per liter of the original bacterial culture. Bacteria were lysed by passing through a French press, and the cell lysate was cleared by centrifugation at 18,000 rpm for 20 min. The supernatant containing the soluble protein fraction was loaded onto a Ni^2+^ loaded HiTrap 5 ml affinity column (GE Healthcare), washed with 2 column volumes of high salt buffer (20 mM NaP_i_ pH 7.4, 1.5 M NaCl, 10 mM imidazole) and eluted with high imidazole buffer (20 mM NaP_i_ pH 7.4, 50 mM NaCl, 0.5 M imidazole) using a linear gradient of 15 column volumes. The GAP-43 containing fractions were collected and the buffer was exchanged by dialysis in the measurement buffer, 20 mM NaPi pH 7.4, 50 mM NaCl and subsequently concentrated to 0.5 mM in an Amicon Ultra-15 centrifugal filter device 10 K NMWL (Amicon).

### NMR experiments

The backbone ^1^H, ^13^C and ^15^N resonances were assigned using a set of high dimensionality experiments exploiting sparse random sampling of indirectly detected time domains, in order to increase resolution. A 3D HNCO and 3D CACON experiments were used as a base spectra for sparse multidimensional Fourier transform (SMFT) processing of higher dimensionality experiments (Kazimierczuk et al. 2009). Backbone assignment was achieved using 4D HN(CA)NH (Zawadzka-Kazimierczuk et al. 2010) 5D HN(CA)CONH (Kazimierczuk et al. 2010), (HACA)CON(CA)CONH (Zawadzka-Kazimierczuk et al. 2012b), (H)NCO(NCA)CONH (Zawadzka-Kazimierczuk et al. 2012b) experiments. Side-chain assignments were obtained using 5D HabCabCONH, 5D HNCOCACB (Zawadzka-Kazimierczuk et al. 2012b) and HC(CC-tocsy)CONH experiments (Kazimierczuk et al. 2009; Hiller et al. 2008). All those5D experiments were acquired at 298 K on an Agilent Direct Drive 700 MHz spectrometer using the standard 5 mm ^1^H–^13^C–^15^N triple-resonance probe head. Additionally, to obtain proline residues assignment two directly ^13^C detected experiments: 3D CACON and 5D (HACA)CONCACON (Bermel et al. 2013) were performed using Agilent Direct Drive 2 800 MHz spectrometer equipped with cryogenically cooled 5 mm ^1^H–^13^C–^15^N triple-resonance probe head.

All NMR data sets were processed by multidimensional Fourier transformation using the home written software package (Kazimierczuk et al. 2006, 2009; Stanek and Kozminski 2010; Stanek et al. 2012) (http://nmr.cent3.uw.edu.pl/software). Sampling artefacts from 3D HNCO and 4D HN(CA)NH were removed using cleaner3d (Stanek and Kozminski 2010) and cleaner4d (Stanek et al. 2012) programs, respectively. The resonance assignment was performed using the TSAR program (Zawadzka-Kazimierczuk et al. 2012a). The input data for TSAR was prepared using Sparky software.

### Extent of assignment and data deposition

**Fig. 1:**
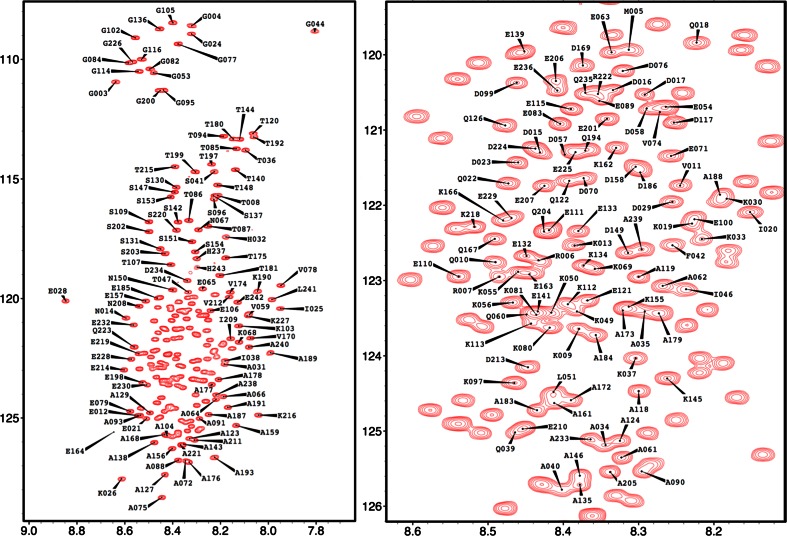
*Left* ^1^H–^15^N HSQC spectrum of human GAP-43 at pH 6 and 298 K. *Right* magnification of the central region of the spectrum

**Fig. 2:**
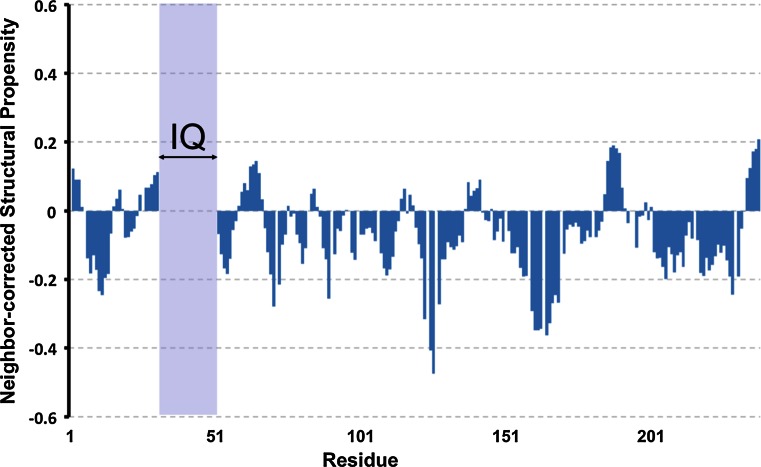
Neighbor-corrected structural propensity index (Tamiola and Mulder 2012) of GAP-43 at pH 6 and 298 K. The IQ domain (from His32 to Leu51), for which only the backbone ^15^N and ^1^HN resonance assignment is available, is highlighted in *blue*

As can be clearly seen from the very narrow peak dispersion in the ^1^H dimension of the ^1^H–^15^N HSQC spectrum (Fig. 1), GAP-43 is an intrinsically disordered protein. Extensive signal overlap in conventional 2D and 3D spectra could be overcome by using the aforementioned 5D experiments. Interestingly, for the full-length protein, the IQ domain (from His32 to Leu51) could not be assigned, probably due to conformational exchange originating from the pronounced helical propensity of this segment. However, the ^1^H–^15^N HSQC spectrum of the IQ domain could be assigned in the context of a shorter construct consisting of the first 59 residues of GAP-43 (GAP-43-NTD) and the assignment transposed to the spectrum of the full-length protein (see supplementary material for full details). However, the side chain assignment of the IQ domain for the full-length protein could not be obtained. Consequently, 98 % of backbone ^15^N, 98 % of ^1^HN resonance have been assigned and, excluding the IQ domain, 89 % of ^13^Cα, 85 % of ^1^Hα, 88 % of ^13^Cβ, 77 % of ^1^Hβ and 97 % of ^13^C′ resonances have been assigned. Additionally, HC(CC-tocsy)CONH spectra allowed the assignment of several side-chain atoms. The neighbor-corrected structural propensity index (Tamiola and Mulder 2012) clearly shows that GAP-43 is devoid of any stable secondary structure element (Fig. 2) but seems to preferentially adopt extended structures (negative index values).

The ^1^H, ^13^C and ^15^N chemical shifts have been deposited in the BioMagResBank (http://www.bmrb.wisc.edu/) under the BMRB accession number 19246.

## Electronic supplementary material

Supplementary material 1 (DOCX 574 kb)
