# Supplementary material for: 1H, 15N, 13C resonance assignment of human GAP-43
Source: Biomol NMR Assign. 2016 Jan 9;10:171–4. doi: 10.1007/s12104-015-9660-9 (PMC4788685; doi:10.1007/s12104-015-9660-9)
Supplement: Supplementary file 1 — Supplementary material 1 (DOCX 574 kb) [file 12104_2015_9660_MOESM1_ESM.docx]

**Supplementary data to:**

^1^H, ^15^N, ^13^C resonance assignment of human GAP-43

Andrea Gabriele Flamm^1^, Szymon Żerko^2^, Anna Zawadzka-Kazimierczuk^2^, Wiktor Koźmiński^2^, Robert Konrat^1^ and Nicolas Coudevylle^1*^

**Cloning and purification GAP-43-NTD**

The GAP-43 N-terminal domain (amino acids 1-59 of the full length protein) was expressed using the vector pETM11 with a N-terminal His6-tag. Expression was done in *E. col*i strain T7 in minimal medium with ^15^N Ammonium chloride and ^13^C_6_ D- glucose as the sole nitrogen and carbon source. The expression is induced at an OD_600_ of 0.8 by adding 0.8 mM IPTG. Expression was carried out overnight (~12h) at 28°C. The cell pellet (after centrifugation at 5000 rpm for 15 min) was resuspended using PBS. Breaking the cells is done by sonication (3 min of 50% amplitude) and the supernatant after centrifugation at 18000 rpm for 20 min was pressed through a 0.45 μm filter before loading it on a Ni2+ -loaded HiTrap 5mL affinity column (GE healthcare). After a washing step with PBS containing 1.5M NaCl, the protein was eluted using 100% HI-PBS (~1-2 column volumes) The tag was cleaved with TEV-protease and the sample dialyzed against the measurement buffer

**Assignment of GAP-43-NTD**

Experiments with ^15^N-^13^C labeled GAP-43-NTD were performed at 298K at a 600Mhz Bruker spectrometer. The NTD construct overlaps nicely with the assignment of the full length protein (Supp. Figure 1), therefore the remaining amino acids (32-53) including the IQ-domain that were not assigned in the full length construct could be identified and assigned using (H)N(CA)NNH-, HNCO- and HNCACB- type of experiments. The neighbor-corrected structural propensity index for GAP-43-NTD clearly shows that in this construct, the IQ domain is devoid of any stable or even transient secondary structure element (supp. Figure 2).


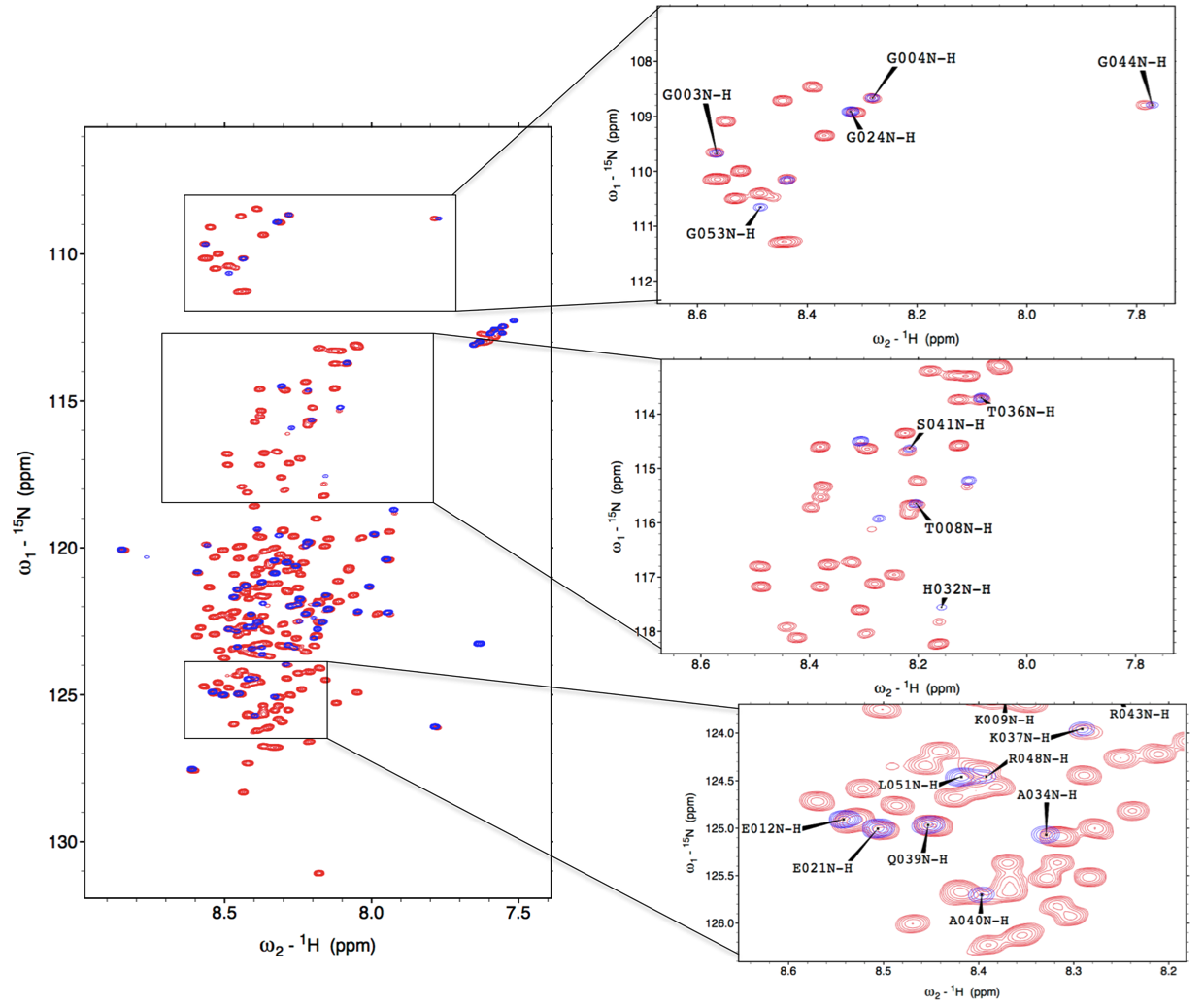


**Supp. Figure 1.** ^1^H-^15^N HSQC spectra of full length GAP-43 (red resonances) and GAP-43-NTD (blue resonances).


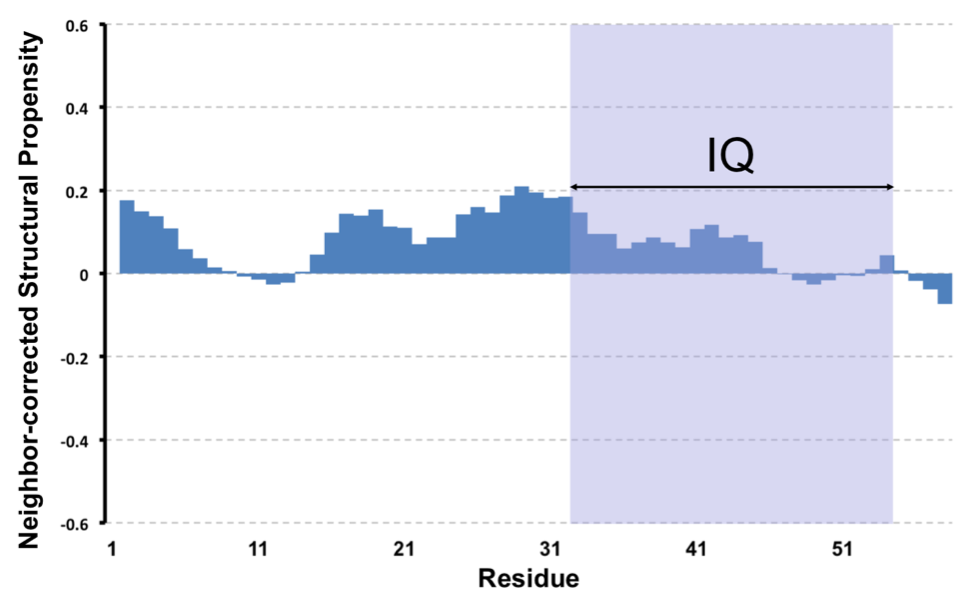


**Supp. Figure 2.** Neighbor-corrected structural propensity index of GAP-43-NTD at pH 6 and 298 K. The IQ domain (from His32 to Leu51) is highlighted in blue.
